# Synthesis of Janus Particles by Seeded Emulsion Polymerization

**DOI:** 10.3390/molecules30183691

**Published:** 2025-09-10

**Authors:** Yingying Wu, Yingchun Long, Guolin Zhang, Qiuhua Wu, Fuxin Liang

**Affiliations:** 1 Liaoning Provincial Key Laboratory for Green Synthesis and Preparative Chemistry of Advanced Materials, Liaoning University, Shenyang 110036, China; 2 Department of Chemical Engineering, Tsinghua University, Beijing 100084, China

**Keywords:** Janus particles, seeded emulsion polymerization, interface, phase separation

## Abstract

Janus particles (JPs), as a special material with anisotropic chemical or physical partitioning, show great potential for application in the fields of material science, biomedicine, energy, and environment. How to achieve fine structural control and large-scale synthesis of JPs is the key point and difficulty for JPs. Seeded emulsion polymerization, as a simple and efficient method, plays an important role in the controlled fabrication of JPs. Here, we provide a comprehensive review of the research progress in the preparation of JPs via seeded emulsion polymerization. We systematically summarize the process mechanisms and key parameters influencing the formation of Janus structures, with particular emphasis on the effects of seed characteristics, polymerization conditions, and component selection on particle morphology and anisotropy.

## 1. Introduction

Janus, the double-faced god of ancient Roman mythology, was depicted with one face looking to the past and the other to the future. As early as 1989, Casagrande et al. used the term “Janus beads” to describe glass microspheres that are hydrophilic on one side and hydrophobic on the other [1]. In 1992, de Gennes, during his Nobel Prize acceptance speech, borrowed the term “Janus” to describe particles with asymmetric structures and distinct chemical or physical partitioning [2,3]. Since then, Janus particles (JPs) have gained significant attention in the academic community.

A representative subclass of JPs exhibit amphiphilic properties similar to molecular surfactants, which originate from their anisotropic dual-surface structure [4,5]. This anisotropic structure not only makes JPs highly efficient in reducing interfacial tension but also enhances the Pickering stabilization effect, significantly improving emulsification ability and interfacial stability [6,7]. As fillers, they provide polymer composites with enhanced interfacial capacitance [8,9], mechanical reinforcement [10], improved toughness [11], and functional tunability [12], among other properties. Thanks to their interesting physical and chemical properties, JPs can be used as emulsifiers [13,14], catalysts [15,16], drug carriers [17], and smart response materials [18].

**Scheme 1:**
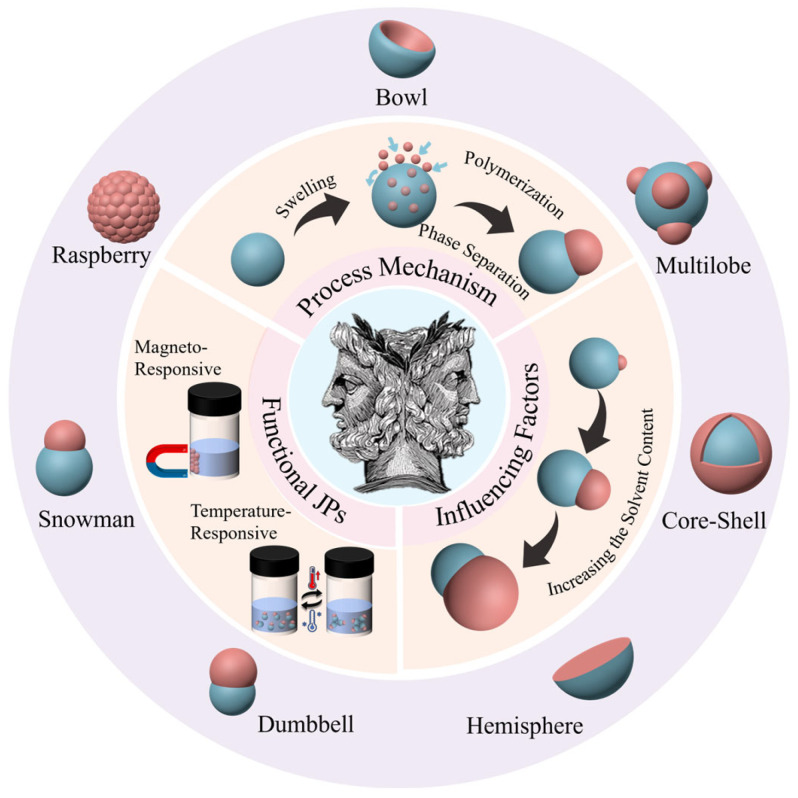
Schematic illustration of different morphologies of JPs prepared by seeded emulsion polymerization.

Over the past decades, various synthetic methods have been developed to produce JPs with different morphologies and functions. These methods include interfacial protection methods [19] (2D planar and 3D Pickering emulsion interfaces), block copolymer self-assembly [20], microfluidics [21], electro-co-injection [22], and seeded emulsion polymerization [23]. The interfacial protection method, while effective at accurately controlling the Janus structure, is challenging for large-scale production [24]. Self-assembly is ideal for producing nanoscale JPs but is hindered by high costs and a limited range of available block copolymer materials. The microfluidic method can prepare JPs with various compositional morphologies, but the size of the prepared particles is often large, and it is difficult to obtain submicron or even nanoscale JPs [21]. Compared with other methods, seeded emulsion polymerization is widely favored due to its ability to mass-produce JPs, the mild reaction conditions it requires, and its capacity to precisely control both the structure and stability of JPs (Scheme 1) [25,26,27,28]. Therefore, this review summarizes recent advances in the synthesis of Janus particles via seeded emulsion polymerization, with emphasis on formation mechanisms, key influencing factors, and material-specific fabrication strategies.

## 2. Fundamentals of Seeded Emulsion Polymerization

### 2.1. Process Mechanism

Seeded emulsion polymerization is a specialized form of emulsion polymerization used to produce JPs. In this method, monomers are dispersed into a dispersion of seed particles through emulsification. The compatibility between the monomer droplets and the seed particles enables the monomers either to diffuse and swell into the interior of the seed particles or to adsorb onto their surfaces. During polymerization, phase separation between the growing polymer and seed material leads to the formation of secondary domains, ultimately resulting in Janus morphology. The reaction proceeds via free-radical mechanisms, including controlled radical polymerization (CRP) techniques, such as reversible addition–fragmentation chain transfer (RAFT) and atom transfer radical polymerization (ATRP). Initiation is typically achieved through the thermal decomposition of chemical initiators (e.g., persulfates) or via photochemical and radiochemical methods, such as UV irradiation or γ-ray exposure [29,30].

### 2.2. Influencing Factors

During seeded emulsion polymerization, the formation of JPs is influenced by a combination of factors [31], including the nature of the seed particles, polymerization reaction conditions, etc.

#### 2.2.1. Seed Particles

**Figure 1:**
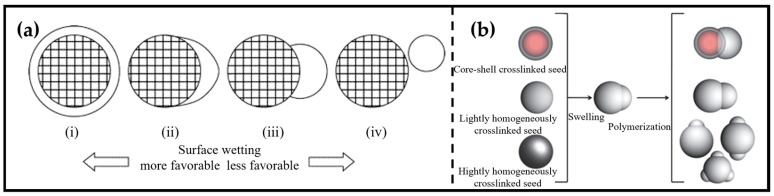
(**a**) Schematic of different states of the monomer—seed particle system as it is (**i**) very favorable, (**ii**) mildly favorable, (**iii**) not very favorable, and (**iv**) not at all favorable for the expelled monomer to wet the cross-linked seed particle surface. Reprinted with permission from Ref. [35]. Copyright 2006 American Chemical Society. (**b**) Schematic diagram showing the formation of the non-spherical homo-phase PMMA particles. Reprinted with permission from Ref. [36]. Copyright 2025 Clearance Center Inc.

In the synthesis of JPs via seeded emulsion polymerization, the final particle morphology and the distribution of phase domains are not only influenced by the size and shape of the seed particles [32,33], but are also significantly affected by their surface properties. Surface characteristics, such as wettability and crosslinking density, play crucial roles in regulating interfacial interactions during polymerization, thereby determining the anisotropic growth behavior and spatial configuration of the resulting phase-separated structures [34]. Mock et al. [35] demonstrated that grafting a hydrophilic polymer layer onto the surface of seed particles can significantly alter monomer wetting behavior and the subsequent polymerization pathway, thereby enhancing the anisotropy of the resulting particles. This hydrophilic grafted layer modulates interfacial tension contrasts, increasing the contact angle between the monomer and the particle surface. As a result, monomer wetting becomes thermodynamically unfavorable, promoting the formation of asymmetrical bulges on the particle surface (Figure 1a). Peng et al. [36] employed monodisperse, crosslinked poly (methyl methacrylate) (PMMA) spheres as seeds. When swollen with methyl methacrylate (MMA) monomer, the resulting particles formed one or more protrusions, depending on the crosslink density of the seeds. In contrast, core-shell PMMA particles consisting of a non-crosslinked core and a highly crosslinked shell as seeds usually yield only single protruding particles (Figure 1b).

#### 2.2.2. Polymerization Reaction Conditions

The morphological evolution of JPs is highly dependent on the systematic modulation of polymerization conditions, including monomer type and concentration [37], polymerization duration [38], surfactant species and concentration [30], as well as solvent properties. These parameters influence not only the swelling behavior and spatial distribution of monomers within the polymer seed but also the driving force and location of phase separation, ultimately determining the anisotropic morphology of the resulting particles.

**Figure 2:**
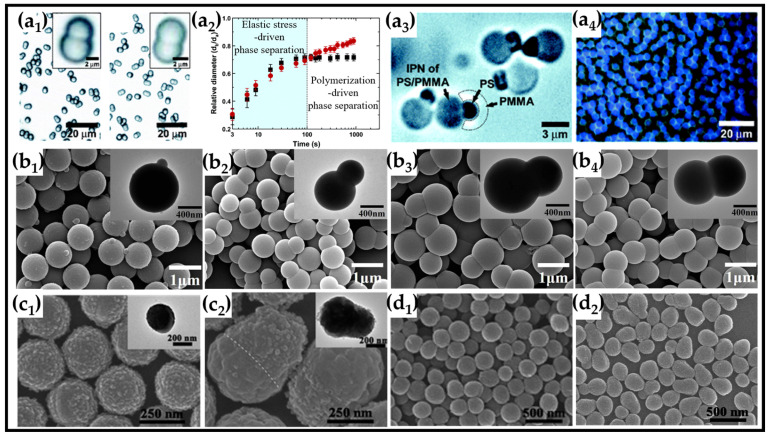
(**a_1_**) Brewster’s Field Microscopy (BFM) images of asymmetrically phase-separated polystyrene (PS) particles obtained at a crosslinked polystyrene/styrene (CPS/St) volume ratio of 1:3, and symmetrically phase-separated PS dumbbell particles formed at a CPS/St volume ratio of 1:4. (**a_2_**) Effect of heating on the diameter, d_b_, of the new bulb, bulb b, relative to the diameter, d_a_, of the seed bulb, bulb a. Experiments were performed both without initiator (■) and with initiator, 0.5 wt% V-65B (●). (**a_3_**) A BFM image of PS (*n* = 1.5894)/PMMA (poly (methyl methacrylate), *n* = 1.4893) dumbbell particles dispersed in a silicone oil (Dow Corning #550, *n* = 1.4945). *n* is the refractive index. (**a_4_**) A fluorescence microscope image of biphasic PS/PBMA (poly (butyl methacrylate)) dumbbell particles (CPS/BMA = 1/2, *v*/*v*). The CPS particles were labeled with 9-vinylanthracene. Reprinted with permission from Ref. [39]. Copyright 2006 American Chemical Society. (**b_1_**–**b_4_**) Morphologies of poly (vinyl benzyl chloride)-poly (3-methacryloyloxypropyltrimethoxysilane) (PVBC-PMPS) particles from different ethanol/water mixtures: ethanol/water mass ratio, *ϕ* = 0.03, *ϕ* = 0.05, *ϕ* = 0.07, *ϕ* = 0.10. Adapted with permission from Ref. [40]. Copyright 2022 Elsevier Inc. (**c**) Some representative anisotropic composite particles by emulsion swelling at varied toluene/particle weight ratio: (**c_1_**) 0.5:1; (**c_2_**) 16.0:1. Reprinted with permission from Ref. [41]. Copyright 2012 American Chemical Society. (**d**) Morphological evolution of the anisotropic composite particles with different sodium dodecyl sulfate (SDS) concentration (mg mL^–1^): (**d_1_**) 0.29; (**d_2_**) 10.00. Reprinted with permission from Ref. [41]. Copyright 2012 American Chemical Society.

The interplay of monomer type, concentration, and polymerization duration profoundly influences the morphological evolution of colloidal particles. A representative example is the work by Weitz et al. [39], who systematically investigated these parameters using crosslinked polystyrene (CPS) particles as seeds in seeded polymerization systems (Figure 2a_1_–a_4_). They demonstrated that initial phase separation is primarily driven by elastic stress induced by monomer swelling. By tuning the ratio of hydrophobic monomers, the anisotropic architecture of the particles can be precisely controlled. As the polymerization proceeds, a mismatch in free volume between the newly formed polymer and the seed particles leads to an increase in interfacial tension, which intensifies phase separation and eventually results in the formation of rigid dumbbell-shaped particles. Moreover, the compatibility between polymers plays a crucial role in structural evolution. For instance, when monomers such as MMA or butyl methacrylate (BMA) are employed, the incompatibility between CPS and the resulting polymers (PMMA or poly (butyl methacrylate) (PBMA)) facilitates phase separation and promotes anisotropic structure formation. This phenomenon is attributed to the poor solvation of the monomers by the seed particles, which inhibits homogeneous monomer diffusion and leads to localized monomer accumulation, inducing asymmetric nucleation and driving further development of anisotropic structures.

The polarity and volatility of solvents dictate the diffusion rate and swelling extent of monomers within the particles, thereby affecting the uniformity of phase separation. To elucidate this effect, Liang et al. investigated how solvent composition modulates the morphology of poly (vinyl benzyl chloride)-poly (3-methacryloyloxypropyltrimethoxysilane) (PVBC-PMPS) composite particles [40]. Monodisperse PVBC seeds were first prepared via dispersion polymerization, and then PVBC-PMPS composite particles were synthesized by seeded emulsion polymerization in ethanol/water mixtures. Since ethanol is less polar and more volatile than water, increasing its content lowers the overall polarity and increases the evaporation rate of the solvent system. As a result, hydrogen bonding between the silanol and the remaining stabilizer polyvinylpyrrolidone (PVP) was weakened, while the hydrolysis and condensation of 3-methacryloyloxypropyltrimethoxysilane (MPS) slowed down, providing sufficient time for the silanol aggregation. These protrusions gradually guided the morphological evolution of the PVBC–PMPS composite particles from core–shell to snowman-shaped and finally dumbbell-shaped structures (Figure 2b_1_–b_4_). In addition, Liang et al. explored the possibility of modulating the morphology of the particles by varying the amount of MPS, type of co-solvent, and pH. By precisely modulating these factors, the precise design and preparation of JPs can be achieved.

During the fabrication of JPs via emulsion-swelling polymerization, surfactants play a pivotal role in stabilizing emulsions, regulating solvent permeation behavior, and controlling morphological evolution. Yang et al. [41] employed an emulsion-swelling strategy to fabricate anisotropic composite particles with tunable Janus balance by extruding the polymer core outward from a core–shell structure to form protrusions (Figure 2c_1_,c_2_). In this process, sodium dodecyl sulfate (SDS) was used to stabilize the oil-water emulsion and to prevent the organic solvent (toluene) from aggregating into large droplets in the aqueous phase. This ensures that the solvent can infiltrate the particles in a controlled manner through the shell. Such stability is critical for the formation of single protrusion JPs. Experimental results showed that in the absence of surfactants or at insufficient surfactant concentrations, protrusion structures fail to form (Figure 2d_1_,d_2_). Therefore, the surfactant serves as a key physical regulator in the formation of Janus morphologies.

## 3. Preparation of Janus Particles

Based on their composition, JPs can be classified into polymer–polymer, inorganic–inorganic, and polymer–inorganic types [42]. These particles may exhibit diverse morphologies, including raspberry [43,44], dumbbell [39], snowman [45], hemispherical [46], multilobed [47], core–shell [48], and bowl-shaped structures [49], with tunable particle size. Inorganic Janus particles are generally divided into two categories: particles composed of distinct inorganic phases and particles with a homogeneous inorganic core but asymmetric surface modifications that result in differences in surface chemistry or physical properties across the two hemispheres. As seeded emulsion polymerization primarily involves the polymerization of organic monomers, it is less suitable for fabricating pure inorganic JPs directly. Therefore, this review focuses on the synthesis of polymer–polymer and polymer–inorganic JPs via seeded emulsion polymerization. Selected examples of functional JPs are also included to illustrate their application potential.

### 3.1. Polymer–Polymer Composite JPs

Polymeric materials, such as polyethylene (PE), polystyrene (PS), PMMA, and PBMA, are widely used in the fabrication of JPs. The selection of these materials not only influences the morphological development of the particles but also critically determines their interfacial properties. By rationally combining polymeric monomers, it is possible to construct surfaces on a single particle that exhibit contrasting physicochemical characteristics, such as hydrophobic and hydrophilic regions, soft and rigid domains, or charged and neutral areas [35,50]. These structural contrasts impart complex functionalities, including amphiphilic interfacial behavior, responsiveness to external stimuli, self-assembly capabilities, and targeted recognition. As a result, such particles hold promise for diverse applications in colloidal stabilization [51], chemical and biological sensing [52], electronic displays, and drug delivery [53].

**Figure 3:**
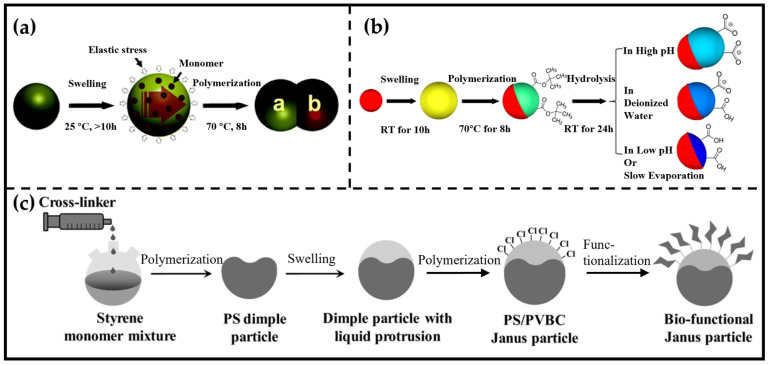
(**a**) Schematic diagram of the preparation of the anisotropic nonspherical dumbbell particles (a: bulb containing most of the original seed particle; b: bulb containing mostly the newly polymerized material). Reprinted with permission from Ref. [39]. Copyright 2006 American Chemical Society. (**b**) Schematic diagram of the preparation of the pH-responsive JPs followed by acid hydrolysis. Reprinted with permission from Ref. [57]. Copyright 2014 American Chemical Society. (**c**) Schematic of the synthesis and click chemistry. Reprinted with permission from Ref. [58]. Copyright 2015 Clearance Center, Inc.

Among commonly used polymer systems, the PS/PMMA combination has been extensively studied due to its thermodynamic immiscibility and differences in interfacial wettability. Seeded emulsion polymerization is often employed to prepare particles within this system (Figure 3a). PMMA exhibits low wettability on PS seed surfaces, and under suitable conditions for interfacial tension and phase separation, well-defined two-phase Janus structures can be obtained [39,54]. Furthermore, replacing rigid polymers such as PMMA with softer materials like poly (butyl acrylate) (PBA), which has a lower glass transition temperature, enables the design of JPs with a gradient in mechanical softness and stiffness [55,56].

Seeded emulsion polymerization enables precise spatial organization of Janus particles through its capacity for controlled phase separation and sequential monomer addition. This capability allows for the selective incorporation of functional monomers into specific domains. The use of functional monomers has enabled the development of JPs with enhanced responsiveness and expanded application potential [50,59]. For example, Lee et al. [57] reported the fabrication of highly uniform, pH-responsive JPs via seeded emulsion polymerization. These particles were composed of a hydrophobic styrene-rich hemisphere and a pH-sensitive acrylic acid (AA)-rich hemisphere (Figure 3b). The AA-containing domain underwent volume changes in response to pH variation, leading to particle shape modulation and altered emulsification behavior. At higher pH values, these particles exhibited reversible transitions between water-in-oil (W/O) and oil-in-water (O/W) emulsion types, driven by changes in amphiphilicity. This pH-adaptive emulsification behavior has enabled their application as responsive solid surfactants.

In addition to Janus systems that rely on the inherent responsiveness of polymer constituents, recent research has proposed an alternative strategy based on chemically modifiable platforms. Unlike poly (acrylic acid) (PAA)-based JPs that respond directly to environmental stimuli, these systems incorporate polymers such as PVBC, which provide reactive sites for subsequent chemical modification. Through efficient coupling reactions such as click chemistry, biological molecules, including sugars, peptides, and nucleic acids, can be selectively grafted onto one hemisphere of the particle, enabling precise spatial functionalization. For instance, Li et al. [58] developed a method that combines seeded emulsion polymerization with thiol-based click chemistry to fabricate biofunctional JPs (Figure 3c). Using thiol–chloride substitution, glucose moieties were covalently introduced onto the PVBC-containing region, thereby conferring the particles with selective recognition capability toward carbohydrate-binding proteins, such as Concanavalin A (Con A). These post-functionalized Janus systems exhibit strong regioselectivity and biological specificity, offering great potential for applications in targeted drug delivery, molecular recognition, and cellular interactions. Table 1 lists the polymer Janus materials prepared by seeded-swelling polymerization.

### 3.2. Polymer-Inorganic Composite JPs

Compared with purely polymeric or inorganic JPs, polymer–inorganic composite JPs combine the multiple advantages of both materials. The polymer component contributes excellent processability, flexibility, and responsiveness to environmental stimuli, while the inorganic component provides outstanding mechanical strength, magnetic properties, and optoelectronic functionalities. This synergy between organic and inorganic phases not only broadens the application scope of JPs but also significantly enhances their performance in practical functional materials. Particularly in the fabrication of functional coatings and flexible films, polymer–inorganic JPs demonstrate strong potential and serve as essential building blocks for advanced devices and multifunctional systems [53]. Depending on the composition of the seed materials, polymer–inorganic JPs can generally be classified into two categories: those originating from polymeric seeds and those originating from inorganic seeds.

#### 3.2.1. Polymeric Seeds

**Figure 4:**
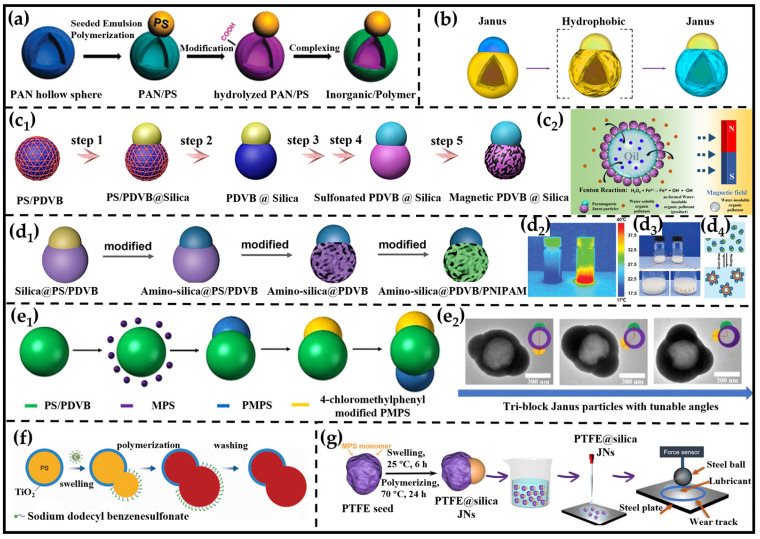
(**a**) Schematic diagram of the preparation of Janus polyacrylonitrile (PAN)/PS colloids. Reprinted with permission from Ref. [61]. Copyright 2010 American Chemical Society. (**b**) Schematic diagram of the preparation of the snowman-shaped Janus composite particles**.** Reprinted with permission from Ref. [45]. Copyright 2015 American Chemical Society. (**c_1_**) Schematic diagram of the preparation of paramagnetic JPs. (**c_2_**) Experimental process route of catalytic degradation. Reprinted with permission from Ref. [62]. Copyright 2024 American Chemical Society. (**d_1_**) Schematic diagram of the preparation of the temperature-responsive JPs, (**d_2_**) infrared thermal image, (**d_3_**) digital photograph, (**d_4_**) illustration of the amino-SiO_2_@poly (divinylbenzene) (PDVB)/poly (N-isopropylacrylamide) (PNIPAM) JPs dispersion at different temperatures. Reprinted with permission from Ref. [63]. Copyright 2023 Wiley-VCH GmbH. (**e_1_**) Schematic diagram of the preparation of the tri-block JPs. (**e_2_**) Schematic diagram of the tri-block JPs with tunable angles. Reprinted with permission from Ref. [64]. Copyright 2018 American Chemical Society. (**f**) Schematic synthesis of anisotropic JPs by emulsion swelling the core–shell structure. Reprinted with permission from Ref. [41]. Copyright 2012 American Chemical Society. (**g**) Schematic of the preparation process of polytetrafluoroethylene (PTFE)@SiO_2_ JPs and friction tests. Reprinted with permission from Ref. [65]. Copyright 2023 The author(s).

In seeded emulsion polymerization, the choice of seed material plays a crucial role in determining the growth behavior and final morphology of JPs. When polymeric seeds are employed, monomers can preferentially swell into the interior or surface regions of the seed particles. During subsequent polymerization, phase separation occurs, resulting in the formation of JPs with spatial asymmetry. Yang et al. [61] synthesized Fe_3_O_4_/PS Janus composite particles using crosslinked polyacrylonitrile (PAN) hollow colloids as seed particles (Figure 4a). A mixture of styrene and divinylbenzene (St/DVB) was introduced into the PAN seeds and polymerized. The elastic mismatch between the PAN matrix and the growing PS domains generated internal stress, which drove partial phase separation and led to the formation of PAN/PS Janus structures. Despite the effectiveness of this approach in creating anisotropic morphologies, the phase separation was often incomplete, especially in systems with high viscoelasticity. This limitation resulted in poorly defined interfaces between the two domains, thereby weakening the compartmentalization of the Janus structure. To address this issue, additional strategies are required to produce JPs with both clear morphological anisotropy and distinct material partitioning. To further enhance structural and functional specificity, the PAN component was hydrolyzed to introduce carboxyl groups (-COOH), which facilitated the selective adsorption of Fe_3_O_4_ nanoparticles onto the PAN region. The resulting Fe_3_O_4_/PS JPs exhibited a well-defined interfacial structure and responded effectively to external magnetic fields, enabling controlled movement of the dispersed-phase droplets.

Achieving clear chemical compartmentalization remains a key challenge in JP synthesis, often requiring post-modification to obtain well-defined structures. To overcome this challenge, Yang et al. propose a new strategy to drive complete phase separation and produce structurally well-defined JPs (Figure 4b). Hollow PS/PDVB particles were used as seeds, and MPS served as the monomer. Through swelling-induced seeded emulsion polymerization, polymer–inorganic composite JPs (PS/PDVB@SiO_2_) were successfully fabricated [45]. During polymerization, phase separation between PMPS and the PS/PDVB matrix led to the formation of SiO_2_ protrusions on the particle surface. This process integrates the hydrolysis and condensation reactions of MPS with polymerization-induced phase separation, enabling complete spatial segregation between the two components and yielding highly defined Janus structures. Moreover, the size of the silica protrusions can be precisely controlled by adjusting the amount of MPS, which in turn allows for tunable surface wettability. As a result, the particles can transition from hydrophobic to hydrophilic behavior and are well-suited for use as solid emulsifiers.

Building upon the synthesized PS/PDVB@SiO_2_ JPs platform, Liang et al. introduced post-synthetic treatments to impart stimuli-responsive functionality (Figure 4c_1_,c_2_). Specifically, snowman-shaped PS/PDVB@SiO_2_ JPs were obtained via seeded emulsion polymerization, followed by selective dissolution of linear PS using *N*,*N*-dimethylformamide (DMF). Distinct chemical functionalities were then introduced: hydrophobic groups were grafted onto one hemisphere, while sulfonic acid groups were anchored on the other. Subsequently, Fe_3_O_4_ nanoparticles were selectively synthesized in situ within the sulfonated domain [62]. The resulting paramagnetic JPs functioned as magnetic colloidal surfactants and were applied in the removal of organic pollutants from wastewater. Notably, these particles achieved removal efficiencies of 98.6% for water-soluble molybdenum (Mo) and 99.9% for water-insoluble *n*-decane.

In addition, Liang et al. proposed a facile method for fabricating thermoresponsive JPs, NH_2_-SiO_2_@PDVB/PNIPAM (poly (N-isopropylacrylamide)), designed for functional coatings with temperature-switchable wettability [63]. In this approach, the SiO_2_ domains of PS/PDVB@SiO_2_ JPs were modified with γ-aminopropyltriethoxysilane (APTES), enabling covalent bonding to substrates such as glass or textile fibers. Following the removal of linear PS, PNIPAM was polymerized within the PDVB cavity (Figure 4d_1_–d_4_). The resulting JPs exhibited reversible wettability behavior: their surfaces were hydrophilic below the lower critical solution temperature (LCST) and became hydrophobic above it. This thermosensitive property makes them attractive for applications such as sweat-responsive coatings for textiles and smart wound dressings.

To construct particles with increased architectural sophistication, the PMPS protrusions were first hydrophobically modified and then used as seeds in a secondary seeded emulsion polymerization using MPS. This yielded ABC-type JPs with adjustable microstructure (Figure 4e_1_,e_2_). The angular spacing between the two SiO_2_ lobes could be regulated by controlling the eccentricity of the PS/PDVB hollow core [64]. The resulting JPs exhibited distinct chemical zoning, demonstrating the potential of this approach for constructing hierarchically structured multifunctional particles.

Apart from the previous methods, a distinct approach was reported by Yang et al. and other researchers, who employed core–shell particles in emulsion-swelling polymerization to achieve directional growth of polymeric lobes [41,66,67]. In this approach, the polymer core undergoes selective swelling and extrudes through the rigid inorganic shell, giving rise to anisotropic structures (Figure 4f). The resulting polymeric protrusions exhibit chemical and interfacial properties distinct from those of the inorganic domain, thereby establishing a reliable platform for constructing amphiphilic and functionally compartmentalized JPs in a structurally precise manner.

Beyond classical stimuli-responsive designs, recent efforts have focused on integrating dynamic functional behavior into static Janus structures for complex environments [68,69,70]. Liang et al. recently developed polytetrafluoroethylene@SiO_2_ (PTFE@SiO_2_) Janus nanoparticles (JNs) for water-based lubrication systems [65]. Using hydrophobic PTFE as seed particles and MPS as the monomer, amphiphilic JPs were synthesized via phase separation (Figure 4g). This approach resolved the issue of the poor aqueous dispersibility of traditional PTFE. PTFE provided lubrication through the formation of a tribofilm on the frictional interface, while the rigid SiO_2_ domains contributed to improved wear resistance. Furthermore, the combination of PTFE tribofilm formation and SiO_2_ reinforcement significantly enhanced the overall lubrication performance. These findings highlight a new strategy for designing Janus structures that not only respond to external stimuli but also offer enhanced stability and performance under complex service conditions. Embedding responsiveness into structurally defined JPs paves the way for advanced applications in tribology, energy systems, smart coatings, and beyond. Table 2 lists the polymer–inorganic Janus materials prepared by seeded-swelling polymerization using polymer seeds.

#### 3.2.2. Inorganic Seeds

**Figure 5:**
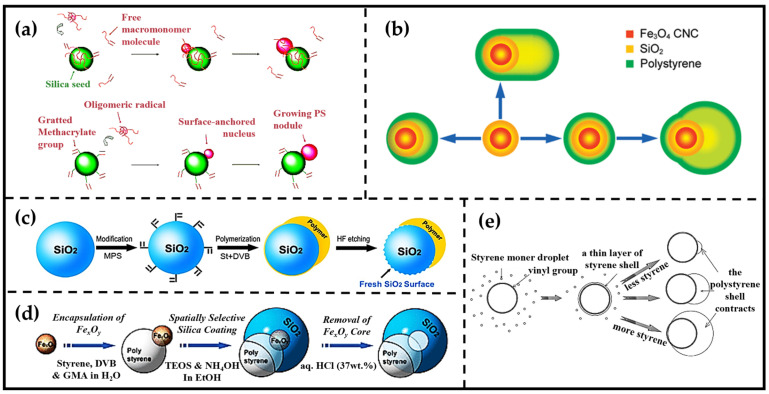
(**a**) Comparison of both synthetic routes to obtain dissymmetrical particles from SiO_2_ particles:  physico-chemical association with a macromonomer (top) and covalent grafting onto functionalized SiO_2_ (bottom). Reprinted with permission from Ref. [71]. Copyright 2005 American Chemical Society. (**b**) Synthesis scheme for Fe_3_O_4_@SiO_2_@PS composite colloids. Reprinted with permission from Ref. [74]. Copyright 2007 American Chemical Society. (**c**) Schematic synthesis of Janus colloids by wet etching the anisotropic composite colloids formed by polymerization-induced dewetting. Reprinted with permission from Ref. [30]. Copyright 2010 The Royal Society of Chemistry. (**d**) Synthesis of the mushroom-shaped Janus particle. Reprinted with permission from Ref. [75]. Copyright 2010 American Chemical Society. (**e**) Scheme for the coating process. Reprinted with permission from Ref. [76]. Copyright 2011 Elsevier Inc.

Compared with polymeric seeds, inorganic nanoparticles, such as SiO_2_, titania (TiO_2_), and magnetic nanomaterials, typically require surface modification to improve interfacial compatibility with polymer phases. In these systems, polymerization of the monomer generally takes place at the surface of the inorganic particles. The difference in interfacial tension between components plays a crucial role in driving morphological development. Reculusa et al. [71,72,73] enhanced the deposition of polymers on SiO_2_ surfaces by either adsorbing macromolecules containing polyethylene oxide segments or by covalently grafting functional trialkoxysilane molecules onto the surface (Figure 5a). These strategies promoted the ordered growth of PS nodules. The ratio between the number of SiO_2_ seeds and the growing PS domains is critical in determining the final structure of the composite particles. When this ratio approaches one to one, asymmetric structures, such as dumbbell-shaped or snowman-shaped morphologies, are frequently observed. Magnetic Fe_3_O_4_ cores coated with a SiO_2_ shell can serve as seeds for the surface polymerization of styrene (Figure 5b). This results in the formation of Fe_3_O_4_–PS hybrid particles with either spherical or anisotropic geometries [74]. The magnetic core may be located centrally or off-center within the polymer shell. However, during polymerization-induced surface dewetting, only a thin polymer layer may form on part of the SiO_2_ surface. Although the resulting particles exhibit structural asymmetry, the absence of clear compositional separation prevents them from displaying true amphiphilic behavior. Therefore, they do not fully meet the criteria of JPs.

To achieve particles with authentic Janus features, Yang et al. [30] proposed a selective etching strategy (Figure 5c). A dilute solution of hydrofluoric acid (HF) or ammonium fluoride (NH_4_F) is used under ultrasonic conditions to remove the thin polymer layer from the SiO_2_ surface. This process exposes a fresh hydrophilic region rich in silanol groups, while the thicker polymer shell remains attached to the opposite side. The resulting asymmetric hybrid particles preserve their original shape and exhibit good dispersibility in both polar and nonpolar solvents. These features conform to the strict definition of JPs, which requires both structural and chemical anisotropy. The exposed silanol-rich surface also allows for further functionalization, enabling the preparation of JPs with customized chemical properties. This approach is scalable and suitable for producing large quantities of structurally well-defined Janus materials. For example, by selectively growing a SiO_2_ hemisphere on the FeₓO_y_ side of FeₓO_y_/polymer snowman-shaped particles and tuning its size by adjusting the amount of silane precursor, followed by the removal of the FeₓO_y_ core through chemical etching, mushroom-shaped hollow SiO_2_/polymer Janus structures can be obtained (Figure 5d) [75]. These particles show strong potential in targeted drug delivery and heterogeneous catalysis. Furthermore, composite JPs composed of SiO_2_, FeₓO_y_, and poly (styrene-co-divinylbenzene) can effectively stabilize oil-in-water emulsions. Each hemisphere can be independently functionalized, making these particles excellent candidates for constructing intelligent interfacial systems with directional transport capabilities. This functionality is particularly promising for applications in phase-transfer catalysis.

In addition, Jia et al. [76] developed a straightforward and efficient one-step emulsion polymerization method to synthesize highly monodisperse SiO_2_ hybrid nanospheres with surface-exposed vinyl groups (Figure 5e). This approach eliminates the need for post-synthesis modification. These nanospheres can be used as seeds to produce uniform JPs and bowl-shaped nanostructures with tunable geometry through aqueous polymerization. These novel structures offer great potential in the development of optically anisotropic materials and nanoscale bioreactors, which are of significant interest in the field of photonic materials. Table 3 lists the polymer-inorganic Janus materials prepared by seeded-swelling polymerization using inorganic seeds.

## 4. Conclusions and Outlook

Seeded emulsion polymerization is an effective method for the preparation of JPs, and a wide range of JPs can be achieved by precisely controlling the seed particles and polymerization conditions. This review has summarized the process mechanisms, key influencing factors, and the successful preparation of polymer–polymer and polymer–inorganic JPs using this method, highlighting its potential for constructing JPs with complex and tunable architectures. The technique offers notable advantages, including scalability, structural stability, and compatibility with functional modifications. However, achieving precise morphological control often requires finely tuned conditions and extended reaction times, which may constrain large-scale production. To overcome these challenges, emerging strategies including computational modeling and intelligent process design offer promising pathways to deepen mechanistic insights and accelerate optimization. Through continued innovation and the integration of such advanced approaches, the productivity and product quality of seeded emulsion polymerization are expected to improve significantly. Furthermore, future research can integrate the structural design and stimulus response properties (e.g., temperature-responsive, magneto-responsive, and pH-responsive) of JPs to broaden their applications in emerging fields, such as flexible electronics and bionic materials.

## Figures and Tables

**Table 1:** Polymer Janus materials via seeded emulsion polymerization.

Composition	Morphology	Property/Application	Ref.
PS/PMMA	Dumbbell Snowman	Colloid surfactants	[39,60]
PS/PBA	Dumbbell Peanut	Amphiphilicity	[23,56]
PS/PNIPAM	Dumbbell	Responsive	[50]
PS/PAA	Hemispherical Spherical Snowman	Colloid surfactants pH-responsive	[57]
PS/PVBC	Sphere	Bioactive	[58]

**Table 2:** Polymer–inorganic Janus materials using polymer seeds.

Composition	Morphology	Property/Application	Ref.
Fe_3_O_4_/PS	Snowman	Magnetic-responsive	[61]
PS/PDVB@SiO_2_	Snowman Dumbbell	Colloidal surfactants	[45]
Fe_3_O_4_/PDVB@SiO_2_	Snowman	Magnetic-responsive	[62]
PNIPAM/PDVB@SiO_2_	Snowman	Temperature-responsive	[63]
PS@TiO_2_	Snowman	Colloidal surfactants	[41]
PTFE@SiO_2_	Snowman	Lubrication	[65]

**Table 3:** Polymer–inorganic Janus materials using inorganic seeds.

Composition	Morphology	Property/Application	Ref.
PS@SiO_2_	Snowman Acorn Bowl Mushroom	Colloidal surfactants	[30,71,75,76]
PS/SiO_2_/Fe_3_O_4_	Spherical Snowman	Magnetic field	[74]

## Data Availability

No new data were created or analyzed in this study. Data sharing is not applicable to this article.
